# Meningitis

**Published:** 1912-03

**Authors:** Floyd S. Crego

**Affiliations:** Attending Neurologist to Erie County, German, Mercy, Emergency Hospital and the Infant Home of Our Lady of Victory. Consulting Neurologist to the Providence Retreat. Buffalo, N. Y.


### BUFFALO MEDICAL JOURNAL

VOL. LXVII. MARCH, 1912. No. 8

### ORIGINAL COMMUNICATIONS

### Meningitis

By FLOYD S. CREGO, M. D.

Attending Neurologist to Erie County, German, Mercy, Emergency Hospital and the Infant Home of Our Lady of Victory. Consulting Neurologist to the Providence Retreat. Buffalo, N. Y.

I have chosen this subject to present to you this evening some points which are of special interest to the surgeon and the general practitioner. Dwelling chiefly on differential diagnosis and the treatment of the various forms which we study.

The first one is that which chiefly interests the surgeon but it also interests the general practitioner because it is so often mistaken for other diseases. I speak of two forms of Pachymeningitis—the chronic external variety and the chronic internal form. Pachymeningitis externa is a slow inflammation of the external layer of the Dura. As you remember the Dura is the periosteum of the internal table of the skull as well as the external covering of the brain. This disease is rather common and is so frequently mistaken for other diseases that it is well worth while to give it a few moments consideration. The cause of this form of meningitis is syphilis, injuries to the head, often very slight—prolonged exposure to heat or cold, prolonged passive congestion. Hence, it is often associated with diseases of the liver. The blood current being interferred with causes a determination of blood to the brain.

The first symptoms of this disease are persistent and localized headache, muscular tremors, frequently epileptiform attacks, confusion of ideas and slight loss of memory—finally insanity. The headache is as I say, localized but frequently it extends over the whole of one side of the head. The pathological condition is a thickening of the Dura and a formation of a membrane between the Dura and the skull. The bones also are thickened and nodes appear on the external surface of the skull. This is one of the useful symptoms in differential diagnosis, namely—the determination of these nodes or a general thickening of the bone which can usually be determined by palpation. Thus, in every case of localized headache it is manifestly of the greatest importance to determine whether these nodes or thickening of the skull is present. This disease as I say, progresses and terminates in mental disease, hence an early diagnosis is essential.

---

Read before the Medical Society of the County of Niagara at Lockport, January 9, 1912.

Diagnosis—it is often mistaken for hysteria, neurasthenia or megrim, as in some cases the severer pain comes on periodically or from time to time. It is also mistaken for epilepsy. In fact, if allowed to continue, it will produce an irritation of the cortex similar to true Jacksonian epilepsy. The headache of hysteria is not so persistent and localized. The headache of neurasthenia is never localized and has the other symptoms common to this disease. The epilepiform attacks are of the petitemal variety often times and muscular weaknesses and muscular tremors are present which is not the case in true epilepsy. The nodes on the skull, when present, and the persistent headache are the chief diagnostic symptoms. The cerebro-spinal fluid may also assist us in diagnosis. When the disease is syphilitic the cerebro-spinal fluid contains lymphocytes. As you know the cerebro-spinal fluid does not normally contain more than one or two lymphocytes to each field but in syphilis of the brain and its membranes we have lymphocytosis That is, from forty to 120 lymphocytes being present in each field. The only other diseases in which these lymphocytes are found are paresis, tabes, herpes zoster, mumps so in the absence of these diseases we can be sure we have to deal with a Pachymeningitis externa if headache, muscular tremors, nodes on the skull and lymphocytosis are present.

The treatment of this disease when it is not syphilitic is surgical. A considerable portion of the skull should be removed. The location of the headache or better yet, the muscular tremor of a certain group of muscles being the guide, the head being trephined over the center controlling the muscles affected. When the disease is syphilitic the hypodermic use of Iodipin first for the prescribed length of time followed by the hypodermic use of the cyanide of mercury is the treatment. The Iodipin is given in doses of 4 c.c. increased to 10 c.c. of the 25% solution of Iodipin, about 40 doses are given—one each day, and usually the headache and other symptoms immediately subside. Iodipin has been found in the saliva and urine from six to nine months after this drug has been discontinued. When we fail to get an immediate result we should resort to the inter venous use of salvarsan two or three doses a month apart being advised. The usual precautions should be followed i.e. that the optic nerve shows no degeneration and the kidneys be in good condition.

Iodipin is one of the remedies which produces great results in the treatment of syphilis of the nervous system and those affections of the nervous system which we designate as tertiary manifestations.

Great care was taken in the experiments with this drug before any announcement was made of it, and the combination seemed to be one of a highly scientific character.

Iodipin was introduced by Winternitz in 1897 but as is usually the case with these drugs, it is just coming into general use, as some of the first reports were not very favorable to the drug as it was not used according to the idea of its discoverer.

Iodipin is a combination of Iodine with the fatty acids of sesame oil whereby it loses its characteristic odor, taste and irritating properties. It is not a mere solution of iodine, but is a chemical compound.

It has been demonstrated by Winternitz that iodipin given either by mouth of subcutaneously, is carried to every tissue of the organism and Iodine could be detected in the abdominal fat and subcutaneous cellular tissues, all the viscera, especially the liver, muscle fibers and bones. The iodine of the iodipin is liberated in the intestines or in the blood being converted into the soluable iodine while the fat is oxidized and acts as a nutrient.

The elimination of iodipin is gradual and continuous and on this account the drug may be administered for a long period in considerable quantities without disturbing digestion or creating any signs of iodism. Doctors Winkler and Stein found that the saliva gave the first reaction usually in 15 to 30 minutes, sometimes 45 minutes, after administration. Dornbluth found iodine in the urine in from 10 to 20 minutes after the first dose of iodipin had been administered and could demonstrate its presence 5 to 10 weeks after the last injection had been given. I saw a case in Jena Germany which had been given the iodipin treatment and no other form of iodine than this for six months previous, which showed the iodine reaction in the saliva and urine. One other note I saw on this subject said that iodine was present one year after treatment by this drug, no iodine having been administered in the meantime and no iodide fats. Iodipin is a combination of iodine with the higher unsaturated fatty acids which are present as glycerides in sesame oil.

The best method of administering iodipin is subcutaneously, being injected into the tissues in the intrascapular region and down the back. Of course the usual antiseptic measures are observed, care being taken that if a large vein is injured another spot is used to make the injection instead of the spot where the vein has been injured. This is accomplished by using a larger needle, introducing it before it is attached to the syringe and observing if there is any large amount of blood drawn which will follow up into the needle. In case no such complication is present the injection is given and usually no abscess follows. In all the cases I have observed there has not been an abscess in a single case. There is said to be a marked reaction to this drug sometimes, the temperature being very high or subnormal. In such cases the drug is discontinued for a short time and then given later. There is very rarely a patient who cannot take this drug and I have only seen one case of iodism and that was in a man who admitted he had had syphilis 8 years ago and who after taking the drug in 4 c.c. doses for a number of times developed a rash which looked very much to me like a scarlet rash. The rash disappeared in a few days, but it was not of the peculiar type of iodide acne. In fact, I have never seen the iodine acne such as we get from potassium iodide, in any given case.

In order to tell whether iodipin given by mouth or hypodermically has been utalized in the body, it becomes necessary to ascertain in what form the iodine is eliminated through the urine, etc. We tested the urine for iodized fats and iodine soaps during iodipin treatment.

These negative results showed clearly enough that human urine excreted during iodipin medication, does not contain fats or soaps, either iodized or uniodized. In other words, iodipin is utilized in the system completely; the iodine is split off and used up, and the fats are fully absorbed.

As I have already stated, the drug is best administered hypodermically although it can also be administered by the mouth. There are two preparations of the drug; a 10% solution for use by the mouth and a 25% solution for hypodermic injections. The dose for the 10% solution is from 4 to 20 c.c. daily. For some time, the 25% could not be obtained but lately it is prepared again by Merck & Co.

Dr. Kindler of the City Hospital at Moabit reports a case of spinal syphilis in which inunction cures and large doses of potassium iodide has been ineffective. Because of the constantly recurring backaches, increasing spasm of the right leg and incontinence of urine the case was treated with iodipin in doses of 2½ drams of the 25% solution injected daily for ten days. After five days the incontinence was stopped and shortly after the spasms and backache also and complete relief of symptoms was brought about after ten days treatment.

The use of mercury may be supplimental to the use of iodipin when thought necessary as is observed by several authors who use it. Where the iodipin is given internally the mercury can be given subcutaneously or by inunction. Where the iodipin is given subcutaneously the mercury is given in the same way in the buttocks. Mercury added to iodipin and injected most always ends in abscess and therefore cannot be employed.

A very interesting case which I have to report this evening was reported to me by a resident physician at the Erie County Hospital, and shows the value of this treatment as well as the value of a lymphocyte count in differential diagnosis. A negro, 30 years of age with symptoms diagnosed as progressive paralysis: tremor of the tongue and indistinct articulation and some mental symptoms, was examined by me. He denied ever having syphilis. The attending surgeon did a lumbar puncture for me. We drew off about 50 drops of the spinal fluid. This was centrifuged for 20 minutes in an ordinary blood certrifuge and a small amount was removed by a pipet and examined under a modified or blind light which showed the presence of from 20 to 30 lymphocytes to each field. This confirmed the diagnosis of cerebro spinal syphilis and we immediately put him on the iodipin treatment and the symptoms rapidly cleared up. I concluded he had a pachymeningitis which was relieved by the treatment. Thus you will see that an apparently hopeless case has apparently completely recovered. It has been some time now since we have heard from the gentleman and I imagine that if he had had a return of the symptoms he would have returned to the hospital. Thus this treatment destroys and absorbes the new formation. To prevent a recurrence of the formation of membrane, mercury should be used. The best form is that of the cyanide of mercury. The 2% solution is used giving from 15 to 30 drops of the drug hypodermically each day for 30 days. To relieve the headache, bromides can be given freely during the treatment in whichever form of treatment is employed, either the surgical or antisyphilitic.

Internal Pachymeningitis is essentially a disease of advanced life. Physiological cerebral atrophy will cause it; frequently seen in persons from 60 to 70 years old, though seen also in younger people. Alcoholism causes 50% of cases; sunstroke and exhausting fevers cause it also.

The symptoms are localized headache, with a feeling of continuous pressure at a certain point. Patients complain of a sensation of something moving back and forth over the brain. There is a slight elevation of temperature, generally not more than 100 to 100.5; the pulse is slow; the respiration is irregular, somewhat of the Cheyne-Stokes order, but not exactly. The pupils are unequal, if the anterior portions be affected. As the membrane increase in thickness we get pressure symptoms and motor disturbance; irregular and spasmodic contractions, partial loss of power in hands and arms, and finally as the paralysis increases complete hemiplegia. The headache gives way to dulness and stupor.

A very characteristic symptom of this disease is the marked remissions; one day you see the patient with a marked hemiplegia, severe headache, elevation of temperature, and the next day he is apparently well. The remission may last a day or two, and the paralysis and headache entirely disappear. This peculiarity is my reason for speaking of this form.

Pathology. The condition which obtains in this disease is the formation of a membrane on the Dura; just exactly how it forms is somewhat doubtful and disputed. Virchow claims that there is first an oozing, then organization, particularly as it is prone to occur in the aged where there is marked cerebral atrophy which lessens the pressure throughout the Dura and giving an opportunity for the blood to ooze out. Others contend that the internal haemorrhage is a primary lesion with subsequent inflamation and formation of a membrane.

We know that as a result of the process, whatever it may be, that there is formed a thick membrane on the surface of the Dura, which may extend over all of one hemisphere or be very limited. It may be thin, or as thick as ⅛ of an inch, it is said even thicker.

The Prognosis is unfavorable; but there are a number of cases where patients have recovered.

The Diagnosis is extremely difficult. It may be mistaken for a slow meningeal haemorrhage; but in meningeal haemorrhage we have coma as a general rule; in this disease we never have coma until late, if at all.

By use of the surface thermometer you will find a local elevation of temperature on the side of the head opposite the lesion. Auscultatory percussion may help you to localize the lesion,

It is also mistaken for leptomeningitis; which we shall consider later, and where the temperature is very high and symptoms very acute.

Treatment of the internal variety is local means, surgical treatment as before; if we have localized headache apply cold and counter irritation at the point indicated. Give considerable doses of ergot, and bromides, later on iodides. Mercury has been recommended but I have never had any success in its use, and rely chiefly on iodides, bromides, ergot and counter-irritation, and what I want to emphasise is surgical interference. It is unnecessary to say that the patient should be kept in bed and on a light diet.

Leptomeningitis, is an inflamation of the arachnoid and pia considered under one head. There are two varieties, *simple* and *tubercular*. If we speak of a patient having meningitis, we usually mean leptomeningitis; another term is cerebral meningitis.

Cerebral leptomeningitis is quite common. The simple form is essentially a disease of adult life, though children are not by any means exempt. In stating that leptomeningitis is quite common, I have followed the German authors but Bramwell says acute leptomeningitis is rare. It must be remembered, he says "That two factors must be present in order that a primary inflamation of the meninges may be produced. The infective organism must be present and the predisposing cause such as trammatism, insolation exposure to cold and alcoholism."

Councilman found in 60 consecutive cases the following organisms; Pneumococcus (18), Meningococcus (21), Streptococcus (18), Staphylococcus (2). In two cases only, the nature of the bacterial infection was undetermined. In many cases the pus may pass from the ear in cases of otitis, following the sheaths of the auditory or facial nerve. In many cases the infective process stops short of the Dura and a serous meningitis may result showing no tendency to become purulent. These cases are favorable for treatment, trephining being resorted to. It is secondary to pneumonia and the acute exanthemata, also typhoid fever.

Symptoms. The initial symptoms are three, *headache, vomiting, fever.* Kernig's sign is present but not absolutely pathognomonic. The disease is often ushered in by a chill, sometimes by convulsions. The fever rises *immediately* to its highest point, and this is a diagnostic symptom. It goes up to 102 or 105. The pulse is very rapid and sometimes the respiratory rate is increased but in most cases the respiration is slow in proportion to the temperature and pulse. This is a differential point from pneumonia. This is usually termed the stage of invasion, lasting from one to 24 hours. Frequently the case will run through all the stages in four or six hours. I have known patients to be perfectly well in the morning and to die at night, having gone through all the stages. Toward the end of the first stage delirium is added to the severe headache; for although delirious he indicates the headache by knitting the brows, clasping or striking the head, moans, cries, etc. During the second stage the temperature remains high, the pulse rapid, breathing about the same, sometimes not at all affected, again quite rapid. A very common and persistent symptom is an obstinate constipation. Toward the end of this stage you see another very characteristic symptom, retraction of the abdomen, though not as marked as in the tubercular form.

During the delirium the patient has delusions and hallucinations, just as in acute mania. The tongue is heavily furred, but not so characteristically as in typhoid.

During all this time, although the vomiting has kept up, the patient has a ravenous appetite, and will take anything given him, but rejects it mmediately.

We may have other symptoms according to the site of the lesion. If the convex serface be affected the disease is likely to be ushered in by convulsions; if the base be affected we get various paralysis, optic neuritis, strabismus, etc.

Another symptom characteristic of this disease comes on sometimes early, sometimes about the second stage, and that is the tache cerebrale; the skin is very red and if you draw your finger across it, you leave a characteristic white line.

We also have cutaneous eruptions, usually herpetic. There is more or less albuminuria, as a general rule. When the base is affected we have opisthotonus, but not so commonly as in the cerebro-spinal type.

The second stage lasts from two to seventy-two hours; then the third stage comes on. The patient falls into a stupor; temperature falls; the pulse rate lowers; the breathing is of the Cheyne-Stokes variety, especially if the base and bulb be affected; pupils are markedly unequal in some cases; in some, with the base affected, we get marked hemiplegia. The convulsions of the second stage have now ceased, the paralysis becomes more marked, the stupor more and more profound.

On the other hand there is sometimes an interval between the second and third stage, the patient may become rational if aroused, and the friends think he is much improved, but you want to be on your guard, for in an hour or two he will probably sink into a final stupor and coma. The leucocyte count is commonly high although notable exceptions occur. Upon lumbar puncture there is found a turbid fluid containing the specific organisms of the meningitis and many cells, the majority of which are polymorphoneuclears. The chemical examination shows choline in the cerebro spinal fluid that is due to myelin degeneration. Choline is also found in hysteria and other so called functional nervous diseases giving us the notion that these so called functional diseases are after effects of slight inflammatory conditions.

Diagnosis. It has to be differentiated from acute mania. The premonitory symptoms of acute mania are not present. The causes are entirely different; the fever is characteristic. In acute mania it is rarely over 100, and you have delusions and hallucinations at once; in meningitis you have simply delusions during the height of the fever.

Typhoid fever with delirium is very frequently mistaken for meningitis. Keep in mind the history of the case and the fever line; in typhoid there is a gradual rise, with morning remissions; in meningitis it becomes excessive immediately. Do not mistake the typhoid rash for the herpetic eruption of meningitis, in which there may also be a general eruption, but not the characteristic rose colored rash of typhoid. Then you have the blood test for typhoid which will make mistakes almost impossible.

Delirium tremens is often mistaken for meningitis. In alcoholic delirium you have marked hallucinations of sight; very rarely any temperature, if any, slight, perhaps 100 and its presence is irregular; in meningitis it is constant.

Thrombosis of the cerebral sinuses is mistaken for it, especially in children. In thrombosis you rarely have fever until three or four days after the coma; in other words the modus operandi is just reversed; coma, delirium, fever.

Bramwell and others say that meningism or pseudomeningitis is a term applied to a symptom complex met with in the course of some of the infective fevers which cannot be distinguished from the real meningitis from the symptoms alone. In some cases there may be a slight inflamation present, in many there may be a meningeal hyperaemia present and in others the symptoms would appear to be due to the direct effect of a toxin on the brain itself. The true meningitis can be distinguished only by the examination of the cerebro spinal fluid. In the pseudo variety the fluid may emerge under pressure but it is clear and presents normal appearances.

Treatment of leptomeningitis. It consists first, in darkening the room, photophobia being one of the symptoms. Put the patient on a light diet, control the fever to a certain extent by antipyretics; and one of the best is aconite. It works especially well in those cases where there is a high elevation in the morning. Give urotropin 10 to 15 grs. every four hours, the spinal fluid being disinfected thereby. The chief reliance is on urotropin. It is also recommended that the blood be examined bacteriologically and the peculiar infection determined and vaccine treatment be resorted to .

To quiet the patient morphine may be absolutely necessary, but bromides are better as they reduce the amount of blood to the brain, while morphine increases it.

The bowels must be kept open by free purgation. When there is a considerable elevation of temperature I give calomel freely, until we get pretty free catharsis. Stimulate the heart as may be indicated.

You know that during the third stage there is an accumulation of fluid within the cranial cavity, pressure, coma, etc. To forestall that, always start in early with iodide of potassium; just before the stage of coma give large doses and as coma comes on resort to trepanning and spinal puncture. I have a record of six recoveries out of ten cases by use of the urotropin treatment, some with lumbar punctures some medical treatment alone.

Chronic Meningitis. It is a disease not very commonly met with. The causes are rather indefinite, syphilis perhaps being the most common; also cold, dampness, slight injuries to the head, and alcoholism.

Symptoms, are persistent headache, vertigo, a sensation as if there were a band around the head. These patients are very restless at night, taking short sleep or being quite sleepless. As the disease progresses there is defective articulation. As it is essentially chronic, it comes on in from one to three weeks. There is weakness of the limbs; frequently spasm of a particular group of muscles. The patient notices that the memory is weak, or his friends do for him; there are symptoms of depression, more or less general paralysis. During all this time the temperature does not vary greatly, sometimes an uneven temperature of 100, sometimes a constant temperature of 100, in many cases no temperature at all. Finally there is general paralysis. The disease lasts from two to eight weeks.

The reason I speak of this form, in that it is often mistaken for general paralysis of the insane; but the differential points is the peculiar delusions, exalted ideas, etc., found in paresis. In this disease there are no delusions, though there may be slight delirium toward evening. We do not get the characteristic and marked physical symptoms seen in paresis.

Multiple cerebro-spinal sclerosis is often mistaken for it. In this disease we have marked volitional tremor, and nystagmus, no tremor in chronic meningitis. In the sclerosis there are also the spinal symptoms and epileptiform attacks. In meningitis there are no epileptiform attacks; sclerosis lasts from one to six years; meningitis terminates in as many weeks.

Medical treatment is same as acute form. Antisyphilitic treatment when indicated. During the last years a number of surgeons have trephined and aspirated the cerebral sinuses to relieve the pressure. Lumbar puncture is a well established procedure and should be resorted to when coma comes on.

You will notice I have not spoken of the Wasserman test for cerebro-spinal syphilis because the lymphocyte count is much more certain. The Noquehi test is said to be oversensitive in cerebro-spinal cases, giving positive results when the other tests were negative. I have not spoken of the use of Flexner serum in the various forms which I have described as its use is or should be limited to the cerebro-spinal (spotted fever) variety which I will discuss at a future meeting. Flexner's, Merck's and other serums being used only when the diplococcus intercellularis is present. This form of meningitis is so easily determined by a lumbar puncture and a bacteriological examination or in cases of urgency a smear can be made and the nature of the infection determined in a few minutes, that the serum need not be given as a precautionary measure as recommended by some.

478 Delaware Avenue.

